# Herpesvirus Antibody Response and Occurrence of Symptoms in Acute and Post-Acute COVID-19 Disease

**DOI:** 10.3390/v16101577

**Published:** 2024-10-07

**Authors:** Julia Butt, Julia Simon, Tim Waterboer, Uta Merle

**Affiliations:** 1 Infections and Cancer Epidemiology, German Cancer Research Center (DKFZ), Im Neuenheimer Feld 280, 69120 Heidelberg, Germany; 2 Department of Internal Medicine IV, University Hospital Heidelberg, 69120 Heidelberg, Germany; uta.merle@med.uni-heidelberg.de

**Keywords:** herpesvirus, COVID-19, PASC, serology, fatigue

## Abstract

Knowledge about the underlying causes of the individual occurrence of symptoms during acute COVID-19 disease and during the post-acute sequelae of COVID-19 is limited. In a German COVID-19 follow-up study, we assessed whether elevated antibody responses to herpesviruses were associated with symptom occurrence in acute COVID-19 disease (n = 96 participants) and during 20 months of follow-up (n = 62 participants). Serum samples were analyzed for their antibodies to herpes simplex virus (HSV)-1 and -2, Epstein–Barr virus (EBV), and Cytomegalovirus (CMV) using fluorescent bead-based multiplex serology. The association of herpesvirus antibodies with symptom occurrence (fatigue, fever, dyspnea, decrease in taste, concentration problems) was assessed using multivariate logistic regression models. High EBV antibody levels were significantly associated with a more than fourfold increased odds of experiencing fatigue during acute COVID-19 disease and during follow-up. High CMV antibody levels were significantly associated with a more than threefold increased odds of experiencing concentration problems and a decrease in taste during the follow-up. The HSV-1 and -2 antibody levels were not elevated in the individuals that experienced symptoms. In conclusion, our findings indicate that herpesvirus infections, specifically EBV and CMV infections, might play a role in symptom development during acute and post-acute COVID-19 disease. It remains to be elucidated whether the elevated EBV and CMV antibodies determined in our study are indicators of herpesvirus reactivation.

## 1. Introduction

As of June 2024, there have been over 775 million confirmed cases of SARS-CoV-2 infection worldwide [1]. During the acute phase of COVID-19 disease, infected individuals may experience flu-like symptoms, including fever, a disturbed sense of taste, fatigue, and dyspnea, amongst others. Further, it has become evident that approximately 10% of infected individuals will develop long-term symptoms, posing a major public health concern [2]. The WHO defined tpost-COVID-19 condition as the continuation or development of new symptoms 3 months after acute COVID-19 disease, with these symptoms lasting for at least 2 months. These symptoms, which resemble those during acute infection in part but also include a wide variety of other symptoms, like cognitive dysfunction, may fluctuate in their occurrence/disappearance over months after acute infection [2].

The factors contributing to the individual occurrence of symptoms at the acute stage and the post-acute stage of disease largely remain unknown. Sex, age, and pre-existing medical conditions have been associated with the severity of acute disease but do not completely explain inter-individual differences [3]. Numerous hypotheses on the occurrence of symptoms after acute disease exist, including persistent SARS-CoV-2 virus or antigen reservoirs, changes in systemic immunity, the induction of autoimmunity, and/or the reactivation of latent viruses [4]. The reactivation of latent herpesvirus-1 (HSV-1), Cytomegalovirus (CMV), and Epstein–Barr virus (EBV) infections upon COVID-19 were noted early on during the pandemic, especially in severe cases [5,6,7,8,9,10,11,12]. A few studies have assessed whether the reactivation of herpesvirus infection upon COVID-19 disease also increases the risk of developing post-acute sequelae of COVID-19 (PASC) and consistently found EBV reactivation to be associated, particularly with the occurrence of fatigue [13,14,15,16,17,18,19]. In this context, reactivated EBV may cause the observed symptoms itself, as it is a well-known cause of chronic fatigue syndrome. Other hypotheses for a causal relation between EBV and PASC include perturbations in immune cell subsets due to EBV and/or the induction of autoimmune antibodies [4]. Studies on other herpesvirus reactivations, including CMV and HSV-1, have provided inconclusive results [13,15,16,17,19]. However, CMV is known to exhaust the T-cell immune repertoire, which may affect the immune response to and the outcome of other infections, such as SARS-CoV-2 [20].

Pre-existing herpesvirus infections can be assessed through the detection of antibodies to viral antigens in blood. Herpesvirus reactivation may be further indicated by the appearance of antibodies to viral proteins that are expressed upon the transition from latent to lytic cycles of viral infection. For EBV, one such antigen is early antigen D (EA-D), which is also clinically applied, in addition to the EBV infection markers viral capsid antigen p18 (VCAp18) and Epstein–Barr nuclear antigen-1 (EBNA-1). Multiplex serology allows for an efficient analysis of the quantitative serum antibody levels to multiple antigens in one reaction [21]. Multiplex serology for herpesviruses, including HSV-1, HSV-2, CMV, and EBV, has previously been validated with high sensitivity and specificity in detecting these respective herpesvirus infections [22]. For EBV, the existing antigen panel has since been expanded to include proteins that are described to be expressed during the transition from the latent to lytic EBV cycle, i.e., Zebra (BZLF1), BGLF2, BRLF1, BXLF1, and BaRF1 [23,24,25,26].

To date, there is limited knowledge about the underlying causes of the individual occurrence of symptoms of acute COVID disease and PASC, with PASC especially posing a major public health concern. Utilizing a cohort of COVID-19 patients with long-term follow-up, we aimed to assess whether elevated antibody responses to herpesvirus antigens at the acute time-point of COVID-19 disease were associated with symptom occurrence during acute and post-acute COVID-19 disease.

## 2. Methods

### 2.1. Study Population

All patients treated for acute COVID-19 at the Department of Internal Medicine IV of the University Hospital Heidelberg with symptom onset between February 2020 and April 2020 were invited to participate in this prospective, non-interventional COVID-19 long-term follow-up study (Ethics Committee of University of Heidelberg: reference number: S-546/2020; DRKS00025089). Written informed consent according to the Declaration of Helsinki was obtained from all patients. The inclusion criteria were status after a PCR-confirmed SARS-CoV-2 infection, prior out- or inpatient treatment (with live discharge) for acute COVID-19, and age ≥ 18 years [27].

Of this cohort, n = 96 patients had blood samples and questionnaire data available for the time-point of acute COVID-19 disease and were included in the present study. Our study population partly overlapped (n = 66 patients) with a previously published cohort [27]. The participants with available blood samples from the acute COVID-19 time-point were in part seen at different follow-up time-points for the follow-up questionnaire data, laboratory analyses, and blood biobanking: all 96 patients were seen at 5 months (M5, 20–22 weeks) after acute COVID-19 disease; 66/96 patients were seen at 12 months (M12, 50–54 weeks); and 67/96 were seen at 20 months (M20, 83–92 weeks). The data and blood samples of 62 of these patients were concurrently available at all 4 time-points (in acute COVID-19 disease and 5 months, 12 months, and 20 months after), and these participants were included in the analysis of the occurrence of symptoms related to PASC.

### 2.2. Outcome Assessment

The occurrence of symptoms related to acute COVID-19 or PASC was assessed at the acute and follow-up time-points using a structured, paper-based questionnaire, as described previously [27]. Briefly, the following symptoms were recorded during acute COVID-19 disease: fever, sore throat, vomiting/nausea, diarrhea, a decrease in taste, anosmia, cough, dyspnea, fatigue, headache, vertigo, cold, body aches, and shivering. The follow-up questionnaire additionally included: reduced exercise capacity, concentration problems, sleeping problems, anxiety, palpitations, hair loss, and (only at 12 and 20 months) difficulty finding words. For the current study, we assessed the association of herpesvirus antibody levels with the occurrence of the five most common symptoms in our study population, i.e., fever, dyspnea, fatigue, a decrease in taste, and concentration problems. Except for decrease in taste, these also belong to the WHO definition of PASC symptoms [28]. Fever occurred in only 5 individuals during follow-up and was excluded from the follow-up analyses, while the occurrence of concentration problems was only assessed during the follow-up.

For follow-up symptom occurrence, we created a combined variable that determined whether a particular symptom occurred at least at one of the follow-up time-points assessed: no symptom occurrence versus its occurrence at ≥1 time-point of M5, M12, or M20 post-acute disease.

### 2.3. Serological Analyses

Multiplex serology for herpesviruses was performed as described previously [21,22,23]. Briefly, selected HSV-1 (gG), HSV-2 (mgG unique), EBV (VCAp18, EBNA-1, EA-D, Zebra, BGLF2, BRFL1, BXLF1, and BaRF1), and CMV antigens (pp150, pp52, pp28, and pp65) were recombinantly expressed as glutathione-S-transferase (GST)-tagged proteins [22,23]. The GST-tagged proteins were affinity-purified on glutathione–casein-coated and distinctly fluorescence-labeled polystyrene beads (Luminex Corp., Austin, TX, USA). Serum samples were incubated with antigen-loaded beads at a final serum dilution of 1:1000, and antigen-bound serum antibodies were labeled with a biotinylated goat anti-human IgG secondary antibody (#109-065-098, Jackson ImmunoResearch, West Grove, PA, USA) and streptavidin-R-phycoerythrin (1:750, MOSS Inc., Elk Grove Village, IL, USA). A Luminex 200 Analyzer (Luminex Corp., Austin, TX, USA) distinguished between the bead types and consequently the bound antigens, as well as quantified the amount of bound serum antibodies as the median fluorescence intensity (MFI) of at least 100 beads per type measured. Antigen- and serum-specific background values were subtracted to obtain the net MFI values [21].

Antibody responses to herpesvirus antigens were measured in serum samples obtained at the acute time-point of COVID-19 disease, as well as in those from all follow-up time-points. Since the antibody levels did not change substantially between the acute disease time-point and the follow-up time-points (Supplementary Figure S1), the antibody responses during acute COVID-19 disease were chosen as the exposure variables. The continuous antibody responses by symptom occurrence are shown in Supplementary Figures S2 and S3.

### 2.4. Statistical Analyses

The median MFI for each antigen in our study population during acute COVID-19 disease was used as a cut-off for high versus low antibody responses. We then applied multivariate logistic regression models with adjustment for potential confounders sex (male vs. female); age at disease onset (<60 vs. ≥60 years); severity of acute disease (mild/moderate (WHO scale score of 3 or 4) vs. severe/critical (WHO scale scores of 5–7)); pre-existing medical conditions (<1 vs. ≥1 condition); and CRP level during acute disease (≤5 mg/L vs. >5 mg/L) to estimate the odds ratios (ORs) and 95% confidence intervals (CIs) for the association of high versus low antibody responses with symptom occurrence. Since the median antibody responses to HSV-2 were below the technical minimum of reliable quantitation (<100 MFI), they were excluded from the analysis.

All graphical presentations and statistical analyses were enabled by GraphPad Prism 9 (GraphPad Software, Inc., La Jolla, CA, USA) or SAS 9.4 (SAS Institute, Cary, NC, USA). *p*-values < 0.05 were considered statistically significant.

## 3. Results

### 3.1. Characteristics of the COVID-19 Cohort at the Acute Disease and Follow-Up Time-Points

The COVID-19 patients at the acute disease time-point and the sub-cohort with blood samples available at all three follow-up time-points (M5, M12, and M20) did not differ in their distribution of sex (54% and 51% female, respectively), age (47% and 49% at an age ≥60 years at acute disease onset), severity of disease (38% and 39% with severe/critical disease), pre-existing medical conditions (53% with ≥1 condition for both), or CRP level during acute disease (75% and 74% with >5 mg/L) (Table 1). The most common symptom reported during acute COVID-19 disease was fatigue, occurring in 84% of the participants, followed by fever, in 74% of the participants. The most common symptom reported during the follow-up period was also fatigue (60%), followed by concentration problems (52%).

### 3.2. Association of Herpesvirus Antibody Levels with Symptom Occurrence during Acute COVID-19

**Figure 1:**
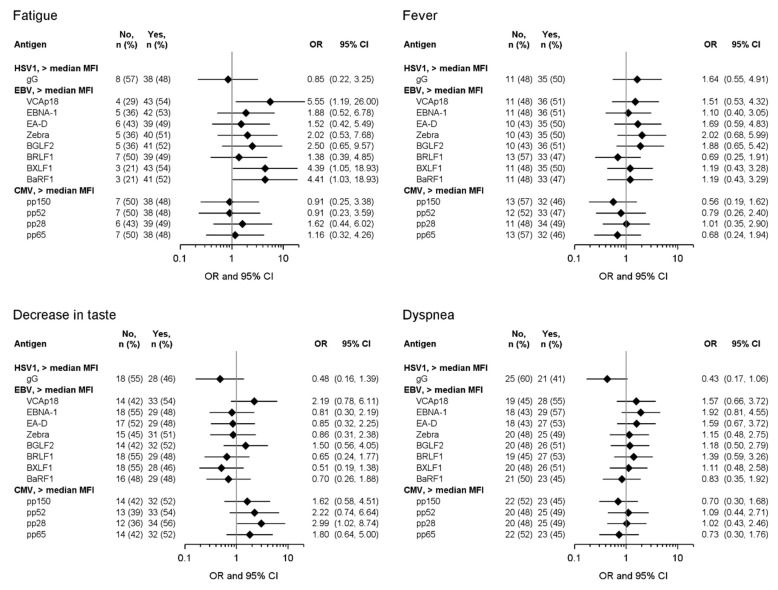
Association of antibody levels above the median MFI to EBV and CMV antigens during acute COVID-19 disease with self-reported symptoms during acute COVID-19 disease (acute cohort, n = 96; a symptom not being present (“No”) versus a symptom being present (“Yes”)). ORs and 95% CIs were estimated using logistic regression models with adjustment for age (<60 vs. ≥60 years), sex (male vs. female), disease severity (mild/moderate vs. severe/critical), pre-existing medical conditions (<1 vs. ≥1 condition), and CRP level at acute disease onset (≤5 mg/L vs. >5 mg/L).

Experiencing fatigue during acute COVID-19 disease was significantly associated with high antibody responses to the EBV proteins VCAp18 (OR: 5.55; 95% CI: 1.19, 26.00), BXLF1 (OR: 4.39; 95% CI: 1.05, 18.93), and BaRF1 (OR: 4.41; 95% CI: 1.03, 18.93) (Figure 1). Furthermore, high antibody responses to the CMV protein pp28 were associated with the occurrence of decrease in taste during acute COVID-19 disease (OR: 2.99; 95% CI: 1.02, 8.74) (Figure 1).

### 3.3. Association of Herpesvirus Antibody Levels with Symptom Occurrence during PASC

**Figure 2:**
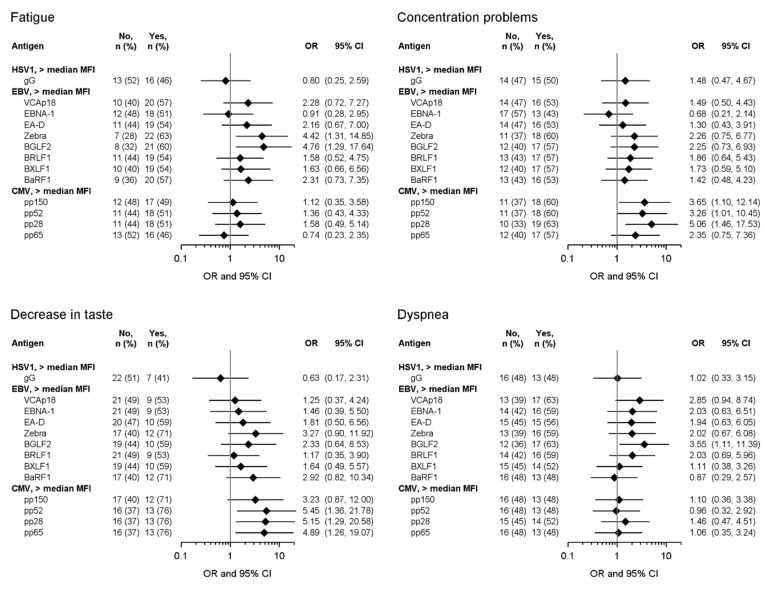
Association of antibody levels above the median MFI to EBV and CMV antigens during acute COVID-19 disease with self-reported symptoms at months 5, 12, and 20 after acute disease (follow-up sub-cohort (n = 62); a symptom not being present (“No”) versus a symptom being present at least in one of the follow-up time-points (“Yes”)). ORs and 95% CIs were estimated using logistic regression models with adjustment for age (<60 vs. ≥60 years), sex (male vs. female), disease severity (mild/moderate vs. severe/critical), pre-existing medical conditions (<1 vs. ≥1 condition), and CRP level at acute disease onset (≤5 mg/L vs. >5 mg/L).

For the symptoms occurring during follow-up, high antibody responses to the EBV protein Zebra were significantly associated with fatigue (OR: 4.42; 95% CI: 1.31, 14.85), and responses to BGLF2 were significantly associated with fatigue (OR: 4.76; 95% CI: 1.29, 17.64) and dyspnea (OR: 3.55; 95% CI: 1.11, 11.39) (Figure 2). High antibody responses to the CMV proteins pp150 (OR: 3.65; 95% CI: 1.10, 12.14), pp52 (OR: 3.26; 95% CI: 1.01, 10.45), and pp28 (OR: 5.06; 95% CI: 1.46, 17.53) were associated with the occurrence of concentration problems. Moreover, high antibody responses to pp52 (OR: 5.45; 95% CI: 1.36, 21.78), pp28 (OR: 5.15; 95% CI: 1.29, 20.58), and pp65 (OR: 4.89; 95% CI: 1.26, 19.07) were associated with the presence of decrease in taste during the follow-up (Figure 2).

## 4. Discussion

In this longitudinal cohort of COVID-19 patients, we demonstrated that high EBV and CMV antibody levels during acute COVID-19 disease were associated with the occurrence of certain symptoms during acute COVID-19 disease and at 5, 12, and/or 20 months post-acute disease.

Fatigue, as one of the most commonly described symptoms during COVID-19 disease, both acute and post-acute, was significantly associated with high antibody responses to EBV proteins. Fatigue is a well-known symptom of EBV infection, and our findings are in line with the current literature, which describes EBV reactivation in PASC patients [13,14,15,16,17,18,19]. Furthermore, high antibody levels to the EBV protein BGLF2 were associated with higher odds of reporting dyspnea during the follow-up. The occurrence of dyspnea during PASC was strongly correlated with the occurrence of fatigue in our study, and the association observed could consequently be a result of this correlation. Thus, this finding warrants further exploration.

Antibodies to the classical EBV reactivation marker EA-D and the infection marker EBNA-1 were not associated with symptom occurrence in our study. The antibody responses measured to these proteins and to VCAp18 were mostly at the saturation level in our study, potentially hindering a proper dichotomization by the median MFI. We added other EBV proteins that have been described to be expressed during the viral transition from the latent to the lytic cycle, i.e., Zebra, BGLF2, BRLF1, BXLF1, and BaRF1 [23,24,25,26]. Supporting the positive associations we observed with these proteins, another recent study identified antibodies to EBV dUTPase BLLF3, also expressed during the lytic viral cycle, to be elevated in patients with chronic fatigue syndrome and patients with PASC [13]. However, it still remains to be verified whether antibodies to the EBV proteins in our multiplex serology assay correlate with clinical EBV reactivation.

High antibody levels to CMV proteins were associated with decrease in taste and concentration problems during acute disease and during the 20-month follow-up of our patients. Peluso et al., 2023, showed that CMV-seropositive individuals were twofold less likely to develop neurocognitive symptoms 4 months after acute COVID-19 disease, contradicting our finding regarding concentration problems [15]. Overall, these findings warrant further verification.

Our study has several limitations and strengths. Firstly, we need to acknowledge that our baseline samples were taken after the onset of acute COVID-19 disease, hindering an analysis of seroconversion or changes in antibody levels to herpesvirus antigens that could indicate reactivation upon SARS-CoV-2 infection. Future studies should consider this in their study design in order to make better assumptions about the timing of the SARS-CoV-2 infection and potential herpesvirus reactivation. We did not observe a substantial change in antibody levels over time during the 20 months of follow-up, further questioning the applicability of antibody levels to herpesvirus proteins as a measure of transient viral reactivation. Thus, reverse causality cannot be excluded, and further experiments that also include herpesvirus DNA detection in blood as a direct marker of reactivation are needed to clarify the question of causality. Another limitation is the small sample size in our study, resulting in a lack of statistical power for multiple testing correction and stratified analyses. Moreover, a larger sample size might have allowed for a more detailed analysis of the longitudinal data with respect to fluctuations in symptom occurrence. The small sample size may have further impacted the generalizability of the results, in addition to the potential selection bias introduced by only including participants that attended at all follow-up time-points. One particular strength of our study is its longitudinal study design. The participants provided data on their symptomology multiple times over the period of 20 months, allowing not only for a one-time assessment but also for consideration of fluctuations in symptom occurrence. Furthermore, we were able to adjust for multiple factors that might have confounded the associations observed, i.e., age, sex, acute disease severity, comorbidities, and inflammatory markers during acute disease. Another strength is our assessment of exposure. The multiplex serology technique allows for a simultaneous quantitative assessment of antibodies to multiple antigens in one reaction.

In conclusion, our study showed that high antibody levels to certain EBV proteins were associated with the occurrence of fatigue during acute and post-acute COVID-19 disease, in line with the findings of previous studies. Future studies should focus on the hypothesis of a potential causal relationship between herpesvirus infections/reactivation and PASC and its mechanism and whether this information could be helpful in terms of prevention and/or treatment of PASC. The other associations observed in this study, i.e., between EBV antibodies and dyspnea, as well as of CMV antibodies with decrease in taste and concentration problems, have not been reported previously and should be confirmed in independent studies.

## Figures and Tables

**Table 1:** Study characteristics of the COVID-19 acute cohort and the follow-up sub-cohort.

Study Characteristics	Cohort at the Acute COVID-19 Disease Time-Point (n = 96)	Follow-Up Sub-Cohort ^c^ (n = 62)
Age at acute disease onset, years, n (%)		
<60	50 (53)	31 (51)
≥60	45 (47)	30 (49)
Missing	1	1
Sex, n (%)		
Male	44 (46)	30 (49)
Female	51 (54)	31 (51)
Missing	1	1
Pre-existing medical conditions at acute disease onset, n (%)		
Asthma	8 (8)	7 (11)
Hypertension	33 (34)	21 (34)
Cardiovascular disease	3 (3)	1 (2)
Diabetes mellitus type 2	6 (6)	5 (8)
Active malignancy	5 (5)	3 (5)
Depression	4 (4)	2 (3)
Obesity (BMI > 30kg/m^2^)	23 (24)	16 (26)
≥1 condition	51 (53)	33 (53)
Acute disease severity, n (%)		
Mild/moderate (WHO scale score of 3 or 4)	59 (62)	38 (61)
Severe/critical (WHO scale scores of 5–7)	37 (38)	24 (39)
CRP levels at acute disease onset > 5 mg/L, n (%)	72 (75)	46 (74)
Immunomodulatory therapy during acute COVID-19 disease, n (%)		
Anti-IL1	2 (2)	1 (2)
Cortisone	1 (1)	1 (2)
Acute symptoms, n (%)		
Fatigue	81 (84)	53 (85)
Fever	72 (75)	46 (74)
Decrease in taste	63 (66)	40 (65)
Dyspnea	53 (55)	34 (55)
PASC symptoms ^a,b^, n (%)		
Fatigue	NA	37 (60)
Decrease in taste	NA	19 (31)
Dyspnea	NA	29 (47)
Concentration problems	NA	32 (52)

^a^ Occurring at ≥1 follow-up time-point (5, 12, and 20 months). ^b^ Due to the low number of individuals reporting fever during the follow-up (n = 3), this symptom was excluded from the PASC analysis. ^c^ Participants with available data at 5, 12, and 20 months after acute COVID-19 disease. CRP, C-reactive protein; NA, not applicable; PASC, post-acute sequelae of COVID-19; WHO, World Health Organization.

## Data Availability

These data are not publicly available due to data protection regulations. Sharing of de-identified data can be requested from the corresponding author.

## Supplementary Materials

The following supporting information can be downloaded at: https://www.mdpi.com/article/10.3390/v16101577/s1, Supplementary Figure S1: Antibody levels to EBV and CMV antigens in the post-COVID-19 sub-cohort (n = 62 individuals) at the acute (0 months) and follow-up (5, 12, and 20 months after acute COVID-19 disease) time-points. Note that antibody responses below an MFI value of 100 are below the lower limit of quantitation (“noise”). Supplementary Figure S2: Antibody response [MFI] to herpesvirus antigens measured at the time-point of acute COVID-19 disease by self-reported symptoms during acute disease (A.-D.). Bars represent the median, boxes the 25th to 75th percentiles, and whiskers the 10th and 90th percentiles. Information on the occurrence of fatigue, fever, and dyspnea was missing for one participant. A Mann–Whitney U-test was performed to assess statistically significant differences in the antibody levels between the groups; ** *p*-value < 0.01. Supplementary Figure S3: Antibody response [MFI] to herpesvirus antigens measured at the time-point of acute COVID-19 disease by self-reported symptoms at months 5, 12, and 20 months after acute disease (A.-D.; a symptom not being present (“No”) versus a symptom being present at least in one of the follow-up time-points) in the follow-up sub-cohort (n = 62). Bars represent the median, boxes the 25th to 75th percentiles, and whiskers the 10th and 90th percentiles. A Mann–Whitney U-test was performed to assess statistically significant differences in the antibody levels between the groups; * *p*-value < 0.05, ** *p*-value < 0.01, and *** *p*-value < 0.001.

## References

[1] WHO Coronavirus (COVID-19) Dashboard. https://covid19.who.int/.

[2] World Health Organization (2021). A Clinical Case Definition of Post COVID-19 Condition by a Delphi Consensus, 6 October 2021.

[3] Jacob L., Koyanagi A., Smith L., Tanislav C., Konrad M., van der Beck S., Kostev K. (2021). Prevalence of, and factors associated with, long-term COVID-19 sick leave in working-age patients followed in general practices in Germany. Int. J. Infect. Dis..

[4] Altmann D.M., Whettlock E.M., Liu S., Arachchillage D.J., Boyton R.J. (2023). The immunology of long COVID. Nat. Rev. Immunol..

[5] Franceschini E., Cozzi-Lepri A., Santoro A., Bacca E., Lancellotti G., Menozzi M., Gennari W., Meschiari M., Bedini A., Orlando G. (2021). Herpes Simplex Virus Re-Activation in Patients with SARS-CoV-2 Pneumonia: A Prospective, Observational Study. Microorganisms.

[6] Seeßle J., Hippchen T., Schnitzler P., Gsenger J., Giese T., Merle U. (2021). High rate of HSV-1 reactivation in invasively ventilated COVID-19 patients: Immunological findings. PLoS ONE.

[7] Simonnet A., Engelmann I., Moreau A.-S., Garcia B., Six S., El Kalioubie A., Robriquet L., Hober D., Jourdain M. (2021). High incidence of Epstein–Barr virus, cytomegalovirus, and human-herpes virus-6 reactivations in critically ill patients with COVID-19. Infect. Dis. Now.

[8] Carneiro V.C.d.S., Alves-Leon S.V., Sarmento D.J.d.S., Coelho W.L.d.C.N.P., Moreira O.d.C., Salvio A.L., Ramos C.H.F., Filho C.H.F.R., Marques C.A.B., Gonçalves J.P.d.C. (2022). Herpesvirus and neurological manifestations in patients with severe coronavirus disease. Virol. J..

[9] Weber S., Kehl V., Erber J., Wagner K.I., Jetzlsperger A.-M., Burrell T., Schober K., Schommers P., Augustin M., Crowell C.S. (2022). CMV seropositivity is a potential novel risk factor for severe COVID-19 in non-geriatric patients. PLoS ONE.

[10] Bernal K.D.E., Whitehurst C.B. (2023). Incidence of Epstein-Barr virus reactivation is elevated in COVID-19 patients. Virus Res..

[11] Brooks B., Tancredi C., Song Y., Mogus A.T., Huang M.-L.W., Zhu H., Phan T.L., Zhu H., Kadl A., Woodfolk J. (2022). Epstein–Barr Virus and Human Herpesvirus-6 Reactivation in Acute COVID-19 Patients. Viruses.

[12] Manoharan S., Ying L.Y. (2023). Epstein Barr Virus Reactivation during COVID-19 Hospitalization Significantly Increased Mortality/Death in SARS-CoV-2(+)/EBV(+) than SARS-CoV-2(+)/EBV(−) Patients: A Comparative Meta-Analysis. Int. J. Clin. Pr..

[13] Liu Z., Hollmann C., Kalanidhi S., Grothey A., Keating S., Mena-Palomo I., Lamer S., Schlosser A., Kaiping A., Scheller C. (2023). Increased circulating fibronectin, depletion of natural IgM and heightened EBV, HSV-1 reactivation in ME/CFS and long COVID. medRxiv.

[14] Klein J., Wood J., Jaycox J., Lu P., Dhodapkar R.M., Gehlhausen J.R., Tabachnikova A., Tabacof L., Malik A.A., Kamath K. (2022). Distinguishing features of Long COVID identified through immune profiling. medRxiv.

[15] Peluso M.J., Deveau T.-M., Munter S.E., Ryder D., Buck A., Beck-Engeser G., Chan F., Lu S., Goldberg S.A., Hoh R. (2023). Chronic viral coinfections differentially affect the likelihood of developing long COVID. J. Clin. Investig..

[16] Zubchenko S., Kril I., Nadizhko O., Matsyura O., Chopyak V. (2022). Herpesvirus infections and post-COVID-19 manifestations: A pilot observational study. Rheumatol. Int..

[17] Su Y., Yuan D., Chen D.G., Ng R.H., Wang K., Choi J., Li S., Hong S., Zhang R., Xie J. (2022). Multiple early factors anticipate post-acute COVID-19 sequelae. Cell.

[18] Gold J.E., Okyay R.A., Licht W.E., Hurley D.J. (2021). Investigation of Long COVID Prevalence and Its Relationship to Epstein-Barr Virus Reactivation. Pathogens.

[19] Apostolou E., Rizwan M., Moustardas P., Sjögren P., Bertilson B.C., Bragée B., Polo O., Rosén A. (2022). Saliva antibody-fingerprint of reactivated latent viruses after mild/asymptomatic COVID-19 is unique in patients with myalgic-encephalomyelitis/chronic fatigue syndrome. Front. Immunol..

[20] Klenerman P., Oxenius A. (2016). T cell responses to cytomegalovirus. Nat. Rev. Immunol..

[21] Waterboer T., Sehr P., Michael K.M., Franceschi S., Nieland J.D., O Joos T., Templin M.F., Pawlita M. (2005). Multiplex human papillomavirus serology based on in situ–purified glutathione s-transferase fusion proteins. Clin. Chem..

[22] Brenner N., Mentzer A.J., Butt J., Michel A., Prager K., Brozy J., Weißbrich B., Aiello A.E., Meier H.C.S., Breuer J. (2018). Validation of Multiplex Serology detecting human herpesviruses 1-5. PLoS ONE.

[23] Simon J., Brenner N., Reich S., Langseth H., Hansen B.T., Ursin G., Ferreiro-Iglesias A., Brennan P., Kreimer A.R., Johansson M. (2022). Nasopharyngeal carcinoma patients from Norway show elevated Epstein-Barr virus IgA and IgG antibodies prior to diagnosis. Cancer Epidemiol..

[24] Feederle R., Kost M., Baumann M., Janz A., Drouet E., Hammerschmidt W., Delecluse H. (2000). The Epstein-Barr virus lytic program is controlled by the co-operative functions of two transactivators. EMBO J..

[25] Littler E., Zeuthen J., McBride A., Sørensen E.T., Powell K., Walsh-Arrand J., Arrand J. (1986). Identification of an Epstein-Barr virus-coded thymidine kinase. EMBO J..

[26] Song L., Song M., Camargo M.C., Van Duine J., Williams S., Chung Y., Kim K.-M., Lissowska J., Sivins A., Gao W. (2021). Identification of anti-Epstein-Barr virus (EBV) antibody signature in EBV-associated gastric carcinoma. Gastric Cancer.

[27] Seeßle J., Waterboer T., Hippchen T., Simon J., Kirchner M., Lim A., Müller B., Merle U. (2021). Persistent Symptoms in Adult Patients 1 Year After Coronavirus Disease 2019 (COVID-19): A Prospective Cohort Study. Clin. Infect. Dis..

[28] Munblit D., Nicholson T., Akrami A., Apfelbacher C., Chen J., De Groote W., Diaz J.V., Gorst S.L., Harman N., Kokorina A. (2022). A core outcome set for post-COVID-19 condition in adults for use in clinical practice and research: An international Delphi consensus study. Lancet Respir. Med..

