# Supplementary material for: Herpesvirus Antibody Response and Occurrence of Symptoms in Acute and Post-Acute COVID-19 Disease
Source: Viruses. 2024 Oct 7;16(10):1577. doi: 10.3390/v16101577 (PMC11512323; doi:10.3390/v16101577)
Supplement: Supplementary file 1 [file viruses-16-01577-s001.zip › viruses-3232355-supplementary.pdf]

SUPPLEMENT

Title: Herpesvirus antibody response and occurrence of symptoms at acute and post-acute COVID-19 disease

Journal: Viruses

Authors and affiliations:
Julia Butt, Julia Simon, Tim Waterboer, Uta Merle

Corresponding author:
Julia Butt
Infections and Cancer Epidemiology, German Cancer Research Center (DKFZ),
Im Neuenheimer Feld 280,
69120 Heidelberg
Germany
j.butt@dkfz-heidelberg.de

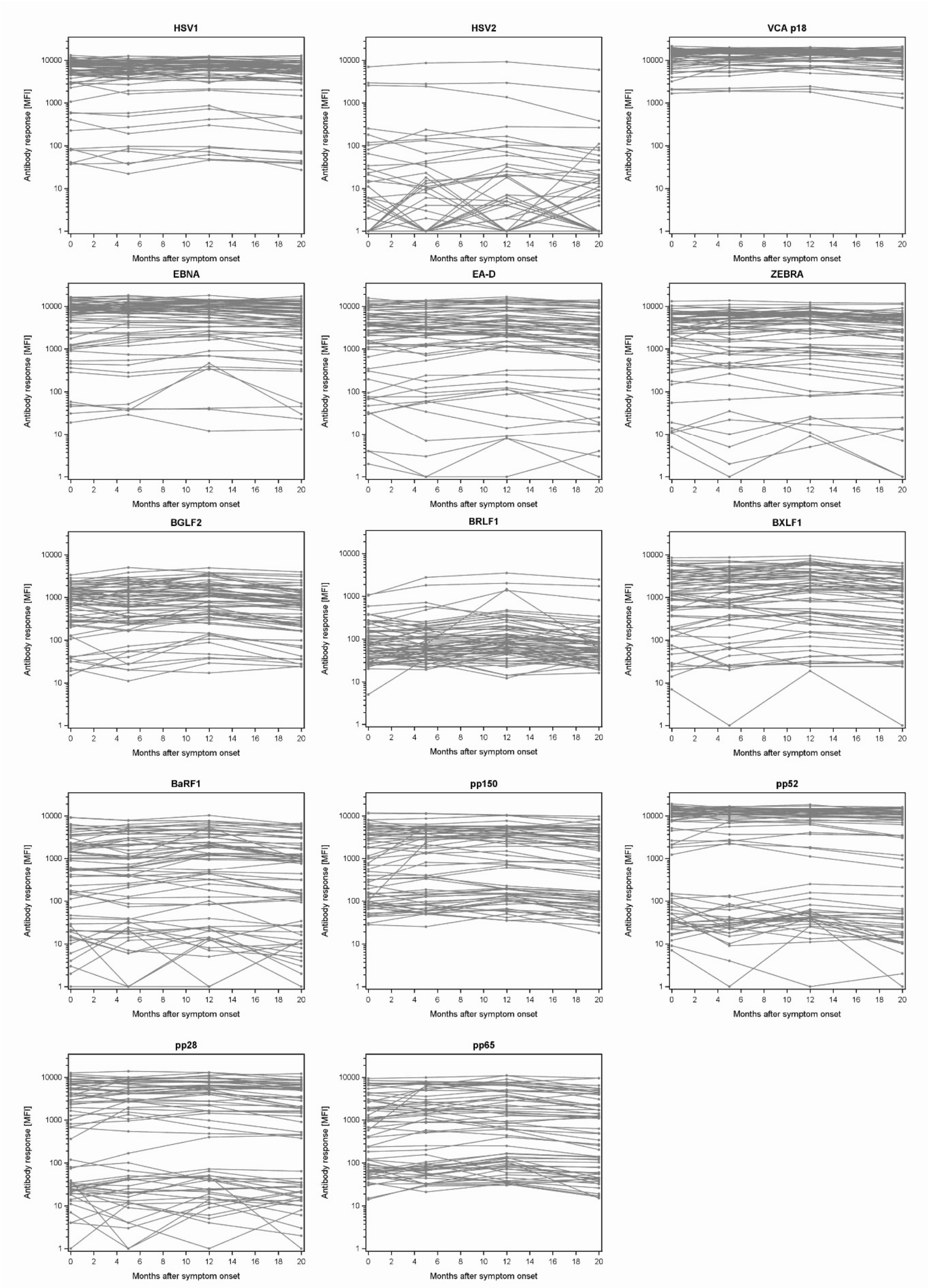

Supplementary Figure S1: *Antibody levels to EBV and CMV antigens in the Post-COVID-19 sub-cohort (n=62 individuals) at acute (0 months) and follow-up (5, 12, and 20 months after acute COVID-19 disease) time-points. Note that antibody responses below 100 MFI are below the lower limit of quantitation ("noise").*

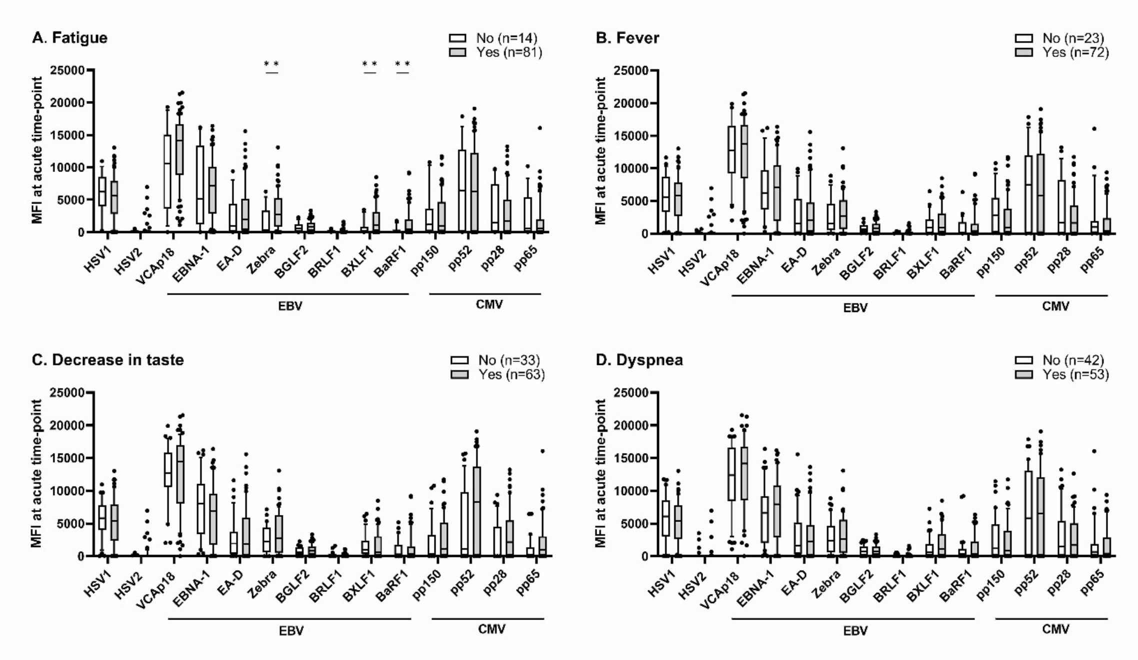

Supplementary Figure S2: *Antibody response [MFI] to herpesvirus antigens measured at the time-point of acute COVID-19 disease by self-reported symptoms at acute disease (A.-D.). Bars represent the median, boxes the 25th to 75th percentile, and whiskers the 10th and 90th percentile. Information on occurrence of fatigue, fever, and dyspnea was missing for one participant. Mann-Whitney-U-test was performed to assess statistically significant differences in antibody level between the groups; **p-value < 0.01.*

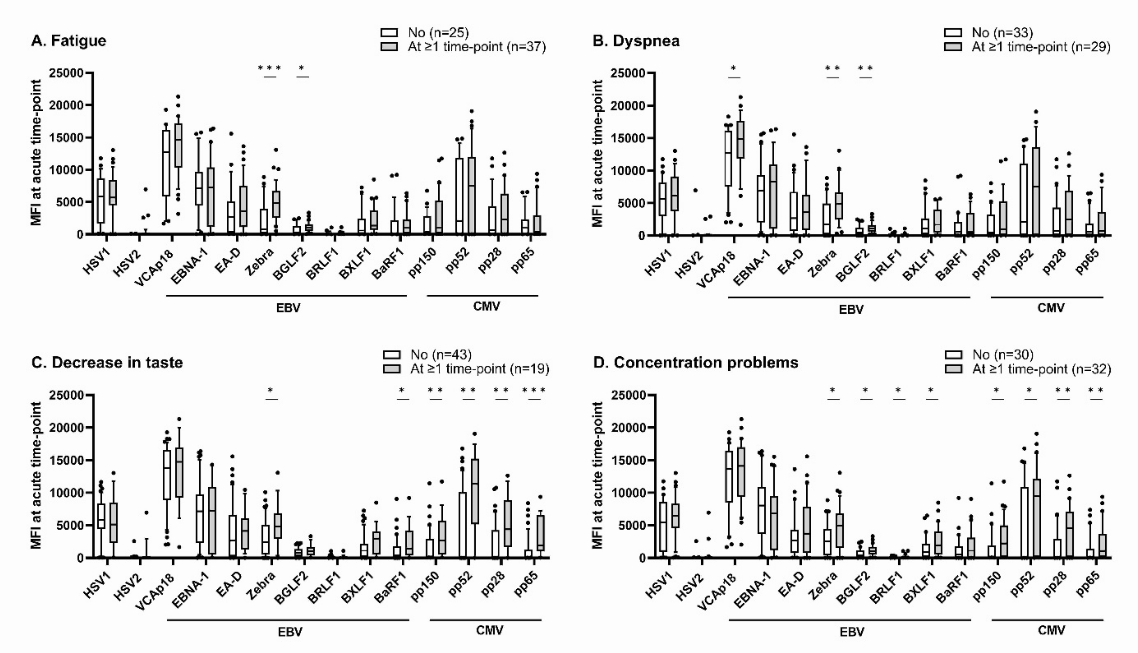

Supplementary Figure S3: *Antibody response [MFI] to herpesvirus antigens measured at the time-point of acute COVID-19 disease by self-reported symptoms at months 5, 12, and 20 months after acute disease (A.-D.; symptom not present ("No") versus symptom present at least in one of the follow-up time-points) in the follow-up sub-cohort (n=62). Bars represent the median, boxes the 25th to 75th percentile, and whiskers the 10th and 90th percentile. Mann-Whitney-U-test was performed to assess statistically significant differences in antibody level between the groups; *p-value < 0.05, **p-value < 0.01, ***p-value < 0.001.*
